# Energetics of Sulfur‐Carbon Interaction

**DOI:** 10.1002/cphc.202200416

**Published:** 2022-11-14

**Authors:** Marie‐Vanessa Coulet, Loïc Gourmellen, Renaud Denoyel

**Affiliations:** ^1^ Aix – Marseille Univ and CNRS MADIREL – UMR7246 Campus de St Jérôme 13013 Marseille France

**Keywords:** adsorption, immersion calorimetry, porous carbon, sulfur, thermodynamics

## Abstract

The energetics of sulfur‐carbon interaction are studied using thermo‐desorption and immersion microcalorimetry experiments. Sulfur is incorporated in meso‐ and microporous carbons by impregnation either from the liquid phase or the vapor phase. Varying the temperature of impregnation enables to fill preferentially microporous domains (vapor impregnation) or both micro‐meso‐macro domains (liquid impregnation). The three carbons lead to similar immersion enthalpies per unit area for liquid sulfur. This suggests that they possess similar surface‐liquid interactions and that liquid sulfur, below the polymerization temperature, wets the whole surface accessible to nitrogen.

## Graphical abstract

**Immersion microcalorimetry** is used to evaluate the sulfur‐carbon interaction. Irrespective of their porosity, the carbons lead to similar immersion enthalpies per unit area for liquid sulfur. This means they have similar surface‐liquid interactions and that liquid sulfur below the polymerization temperature, wets the whole surface accessible to nitrogen.

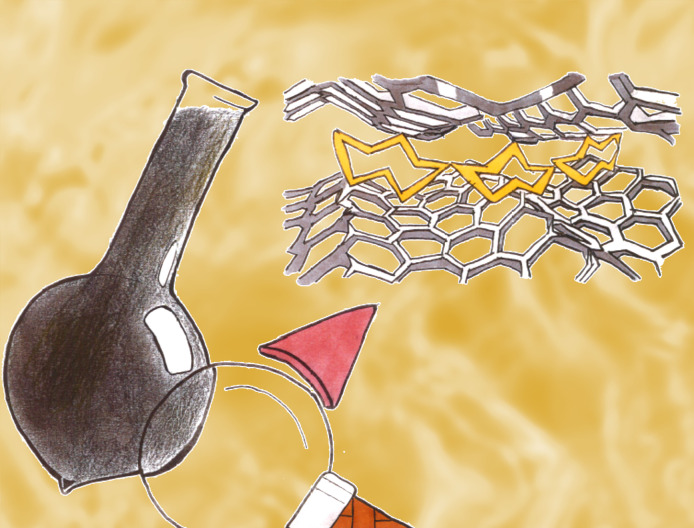

The confinement of sulfur in porous matrices is an active field of research for many years. The first works were essentially fundamental and concerned its confinement in zeolites. At that time, the aim was to identify, essentially by spectroscopic methods, the allotropes (rings or chains) that can be stabilized in the pores.^[1]^ More recently the interest for confining sulfur has increased with the development of lithium‐sulfur batteries.^[2]^ For those applications, sulfur is confined in porous carbon matrices to realize conductive electrodes. The confinement is reported to reduce the formation and the diffusion of lithium polysulfides that are at the origin of the so‐called *shuttle effect*.^[3]^ The efficiency of sulfur‐based electrodes depends on (i) the pore size distribution and organization of the carbon host, which affects the diffusion of electrochemically active species, (ii) the interaction of sulfur with carbon, which has an influence on the long‐term stability of the electrode and (iii) the extent of surface area that is in direct contact with sulfur. It is then clear that it is of utmost importance to choose materials with suited pore size distributions and to control as much as possible the procedure of pore filling since the amount of sulfur in the composite has direct consequences on the gravimetric energy density of the electrode. In terms of filling procedures, sulfur is generally introduced via liquid impregnation,^[4]^ vapor infiltration^[5]^ or even from the solid state *via* the so‐called *spillover* phenomenon.^[6]^ Depending on the chosen temperature, it is excepted that different allotropes are stabilized in the pores which might influence the amount of sulfur that can be encapsulated. In terms of electrochemical efficiency, the sulfur confined into (ultra) microporous carbon becomes inaccessible to carbonate solvent from the electrolyte which reduces considerably the formation and the dissolution of non‐wanted species.^[7]^ The present study focusses on the energetics of sulfur‐carbon interaction, which is not explicitly addressed in literature. This is done here by analyzing thermodesorption curves of samples impregnated by sulfur and by using a new approach based on immersion microcalorimetry. Indeed, immersion calorimetry of porous carbon in water or organic liquids, has up to now only be used to improve their pore size characterization.[^8^, ^9^, ^10^] It is used here for the first time at high temperature and with liquid sulfur in order to assess sulfur accessibility to the pore network.

Three commercial porous carbons are used: a mesoporous carbon (C1) and two microporous carbon labeled C2 and C3. More details are given in SI (section 1). Thermogravimetric analyses and nitrogen sorption isotherms of the carbon matrices before impregnation are shown in Figure 1.

**Figure 1:**
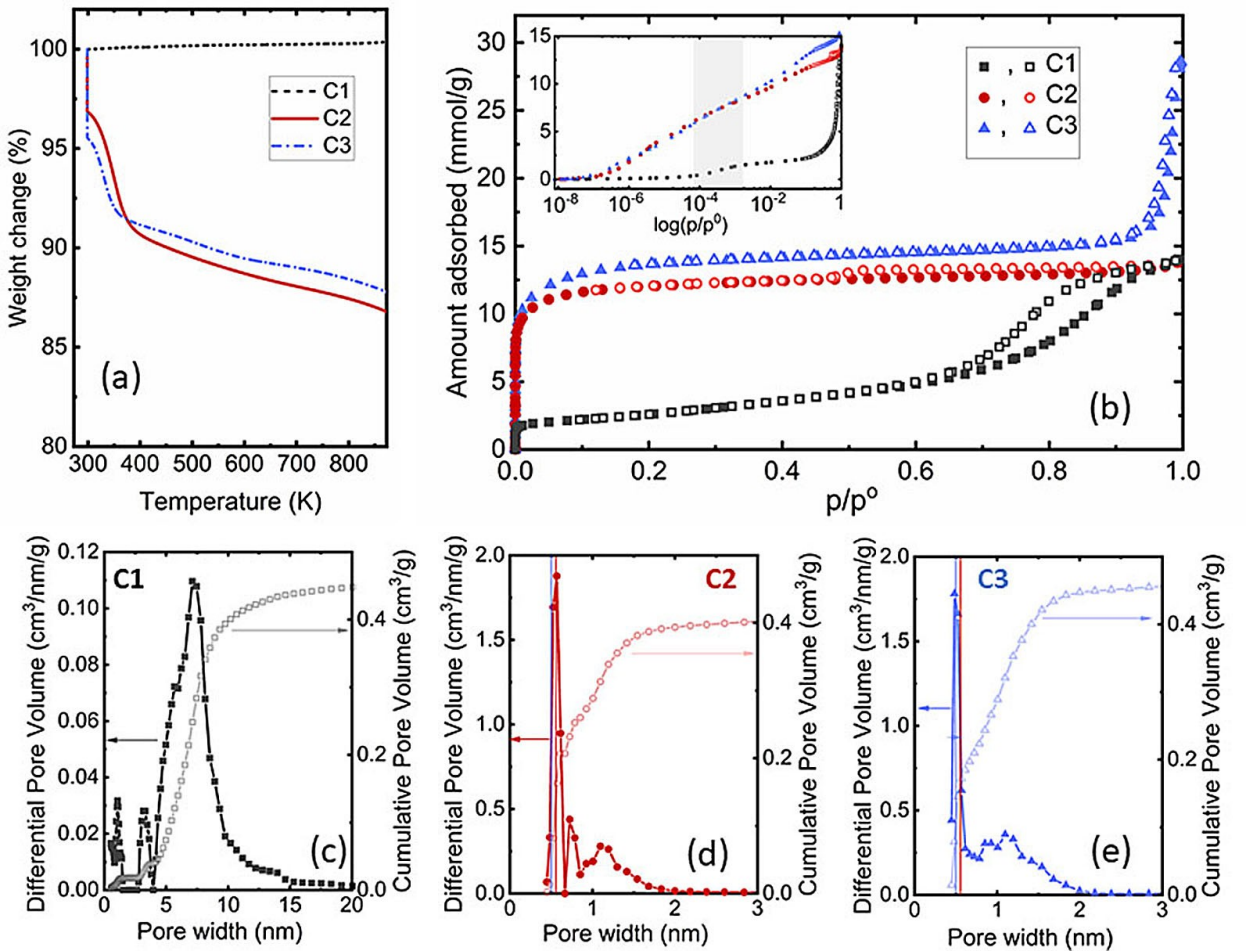
Porous carbons characterization. (a) TGA analyses realized under nitrogen atmosphere. (b) Nitrogen sorption isotherms performed at 77 K. Full symbols are used for the adsorption branch and open symbols are used for the desorption branch. The inset show the isotherm in logarithm scale in order to make more visible the inflexion points. (c–e) Pore size distribution (full symbols) and cumulative pore volume (open symbols) calculated using NLDFT (C1) and using QDFT (C2, C3) on carbon slit pore kernels.^[11]^

The two microporous carbons (C2 and C3) loose around 10 % of their mass between 300 K and 423 K (Figure 1a). This loss can be attributed to the elimination of residual water physisorbed inside the pores. In contrast, the mass remains constant for mesoporous C1 sample suggesting that this carbon is hydrophobic. Water adsorption isotherms (Figure S1 in SI) confirm their different affinity towards water. In the case of nitrogen adsorption, the type‐IV isotherm (Figure 1b) obtained for C1 carbon evidences its mesoporous character. The hysteresis can be assigned to the H1 type^[12]^ since the adsorption and desorption branches are nearly parallel. The adsorption isotherms of C2 and C3 are of type I, typical for microporous samples with high affinity at low pressure. In the case of C2 the closure point of the hysteresis loop is close to a relative pressure of 0.4, indicating that cavitation may occur during nitrogen desorption. This shape of the hysteresis loop is characteristic for samples with ill‐defined pores between plates (H4 type).^[12]^ For C3 sample, a hysteresis is observed at high relative pressures. This is consistent with the co‐presence of large mesopores and confirmed by mercury porosimetry (Figure S2 in SI). In the inset of Figure 1b, the adsorption isotherms are plotted in log scale. The one of C3 starts at slightly lower pressures than that of C2 which means that the distribution of micropore sizes is shifted towards smaller pores if the same surface chemistry is assumed. This is confirmed by the pore size distributions (Figure 1c): the pore size distributions for C2 and C3 are very similar and only differ slightly by the minimum pore width, which is shifted towards smaller values for C3 carbon (see vertical lines in Figure 1c). Mesoporous C1 sample shows a wider pore size distribution with a main contribution around 6 nm. The small contributions at 1 nm and 3 nm are related to the graphitization of the surface which is also responsible for the inflexion points around p/p^0^=8.10^−4^ and p/p°=0.3 on the adsorption isotherm. The equivalent BET surface areas and pore volumes are summarized in Table S1.

Figure 2 presents high‐resolution TEM images and the corresponding EELS spectra for the three carbon matrices.

**Figure 2:**
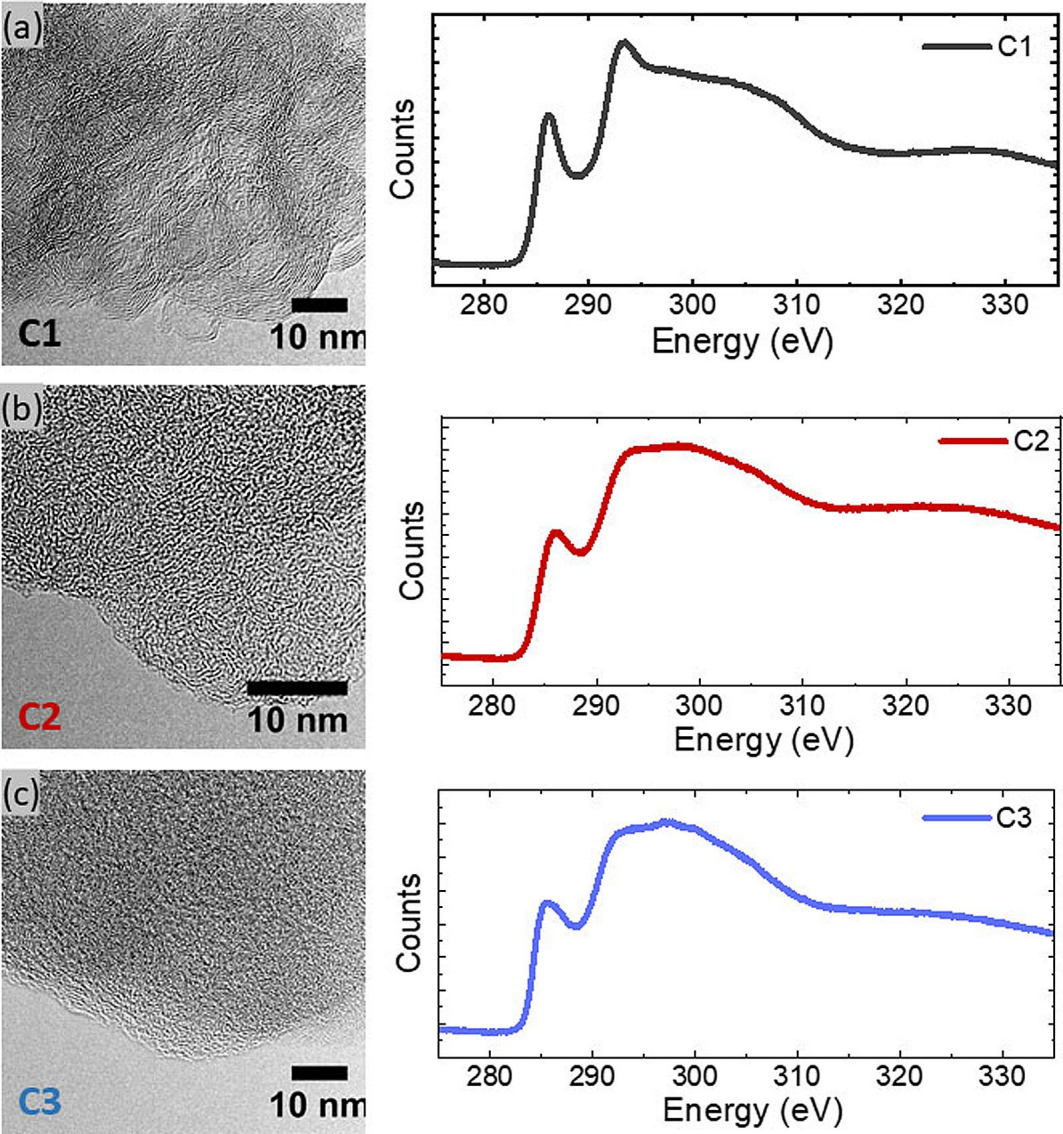
TEM‐EELS characterization of the carbon matrices. The high resolution TEM images (left) are accompanied by the corresponding EEL spectra (right).

The size and the number of stacked graphene layers are the highest for the C1 mesoporous carbon and the smallest for the C3 microporous carbon (Figure 2c). This confirms that the organization of graphene layers is directly linked to the specific surface area.[^13^, ^14^] The EELS spectra recorded in the core loss region all possess a peak around 285 eV, related to the π* antibonding orbitals and typical for sp^2^‐hybridized carbon.^[15]^ Nevertheless, the fraction of sp^2^‐bonded carbon is lower in C2 and C3 carbon since the peak is much less intense in those samples. At last, only the mesoporous C1 presents a clear feature around 292 eV which is characteristic of the unoccupied σ* states. These observations confirm that mesoporous C1 sample is a graphitized carbon while the microporous C2 and C3 are mainly amorphous carbons with a low degree of graphitization.

The carbon matrices were impregnated either from the liquid phase (at 413 K, well below the polymerization temperature) or from the vapor phase (at 873 K). All the details of the procedure are given in SI (section 4). The composites are denoted S_L_@Cx or S_V_@Cx, where x is 1, 2 or 3 depending on the porous carbon matrix and L and V stand for liquid and vapor. As evidenced in Figure 3a and 3b, after impregnation the amount of adsorbed nitrogen is very low for all samples. This indicates that sulfur has either filled or blocked the pores, whatever the sample. As seen in Figure 3b, the mesoporous sample C1 keeps a number of open pores in the case of vapor impregnation, whereas for liquid impregnation the hysteresis loop has disappeared. Since the initial proportion between sulfur and carbon was the same within 1 % (see SI, Table S2), this indicates that the pore filling may be less efficient in the case of vapor impregnation. This is probably linked to an unwanted temperature gradient during the experiment.

**Figure 3:**
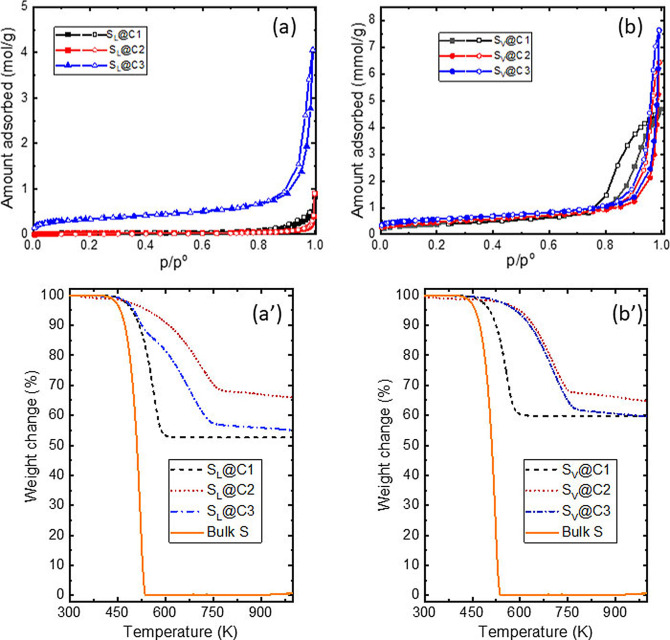
Nitrogen sorption isotherms and thermo‐desorption analyses of the S@C composites synthetized by liquid impregnation (a,a’) or by vapor impregnation (b,b’).

For microporous samples, the onset of a hysteresis loop at p/p^o^ values close to 1 suggests the presence of mesopores. They probably have their origin in the partial filling of macropores by sulfur. This suggests that micropores are preferentially filled and it indicates that, like for any fluid, the affinity of sulfur is larger when the pore size is smaller. The BET surface areas and total pore volumes obtained after impregnation (liquid or vapor) are given in SI (Table S3).

The filling of the pores and the influence of pore size on sulfur affinity for the surface are confirmed by the thermo‐desorption analyses. The desorption curves of pure sulfur are compared with the ones of liquid (Figure 3a’) and vapor (Figure 3b’) impregnated samples. For pure sulfur the temperature range of evaporation is narrow since it is a pure element, even if it may contain several types of molecules (rings, polymers). For impregnated samples, it is observed that the temperature of sulfur desorption is shifted towards higher temperatures when the sulfur is confined inside the pores and this shift is higher as the pore size decreases. The desorption of sulfur is observed between 430 K and 633 K for C1 and between 440 K and 795 K for C2 and C3. A narrow range of temperature departure is observed for C1 which possesses mainly mesopores. In contrast, C2 and C3 have larger pore size distributions since they contain micropores, mesopores and even macropores. These results confirm that sulfur really fills the pores for both impregnation methods. Indeed, if it was outside the pores and simply blocking them, the temperature of desorption would be more or less the same as the one of pure sulfur evaporation. The weight losses obtained by thermo‐desorption (SI, Table S2) give systematically lower pore filling than the one initially targeted, particularly for the impregnation from the vapor phase which confirms the results obtained by adsorption. For mesoporous carbon C1, both liquid and vapor impregnation lead to a similar desorption temperature, whatever the pore filling. This tendency is not the same for microporous carbons. The impregnation from vapor at 873 K leads to an important increase of the desorption temperature. This could mean that under those conditions, smaller molecules can be formed and they are preferentially adsorbed in the micropores. In the case of liquid impregnation, the range of temperature desorption is larger. For instance, in the case of C3, the desorption curves follow initially the one of C1 and then join the temperature range observed for vapor impregnation. This two‐regimes behavior may indicate that both micropores and larger pores (meso or macro) are filled by liquid sulfur. Finally, one can conclude that (i) sulfur confined in micropores is more strongly retained than in the one in mesopores, (ii) it is possible to fill the samples either from the liquid or the vapor phase and (iii) vapor impregnation leads to a more homogeneous filling in the micropores. Nevertheless, these observations are based on *ex‐situ* experiments where the dynamic of filling and the interaction energy between sulfur and surface cannot be quantified since thermo‐desorption curves are a combination of kinetics and thermodynamics aspects.

To the best of our knowledge, there is no attempt in the past to evaluate experimentally the interaction between sulfur and carbon surfaces. The details of the experimental method are given in SI (section 5). The calorimeter temperature was set to 413 K *i. e*. at a temperature above the melting point of sulfur but below its polymerization transition. At this temperature, liquid sulfur mainly consists in S_8_ rings and has a low viscosity. A first interesting result of the calorimetry approach, directly accessible from the raw data, is the possibility to follow the kinetics of pore filling. As seen on the thermograms in Figure 4, the phenomenon is relatively fast since the baseline of the calorimetric peak is recovered after 1500 s whatever the sample. This has to be compared to the 1000 s needed for a simple heat effect such as the introduction of a piece of alumina to calibrate the system (see inset in Figure 4a). This means that the wetting of carbon by liquid sulfur is achieved within a few minutes at this temperature where the viscosity of sulfur is low (∼6.10^−2^ Pa. s^[16]^). By contact angle measurements, the spontaneous wettability of carbon by sulfur was already studied using sessile drop method^[17]^ but the observed time for complete infiltration of sulfur is larger than the one obtained here by the immersion method.

**Figure 4:**
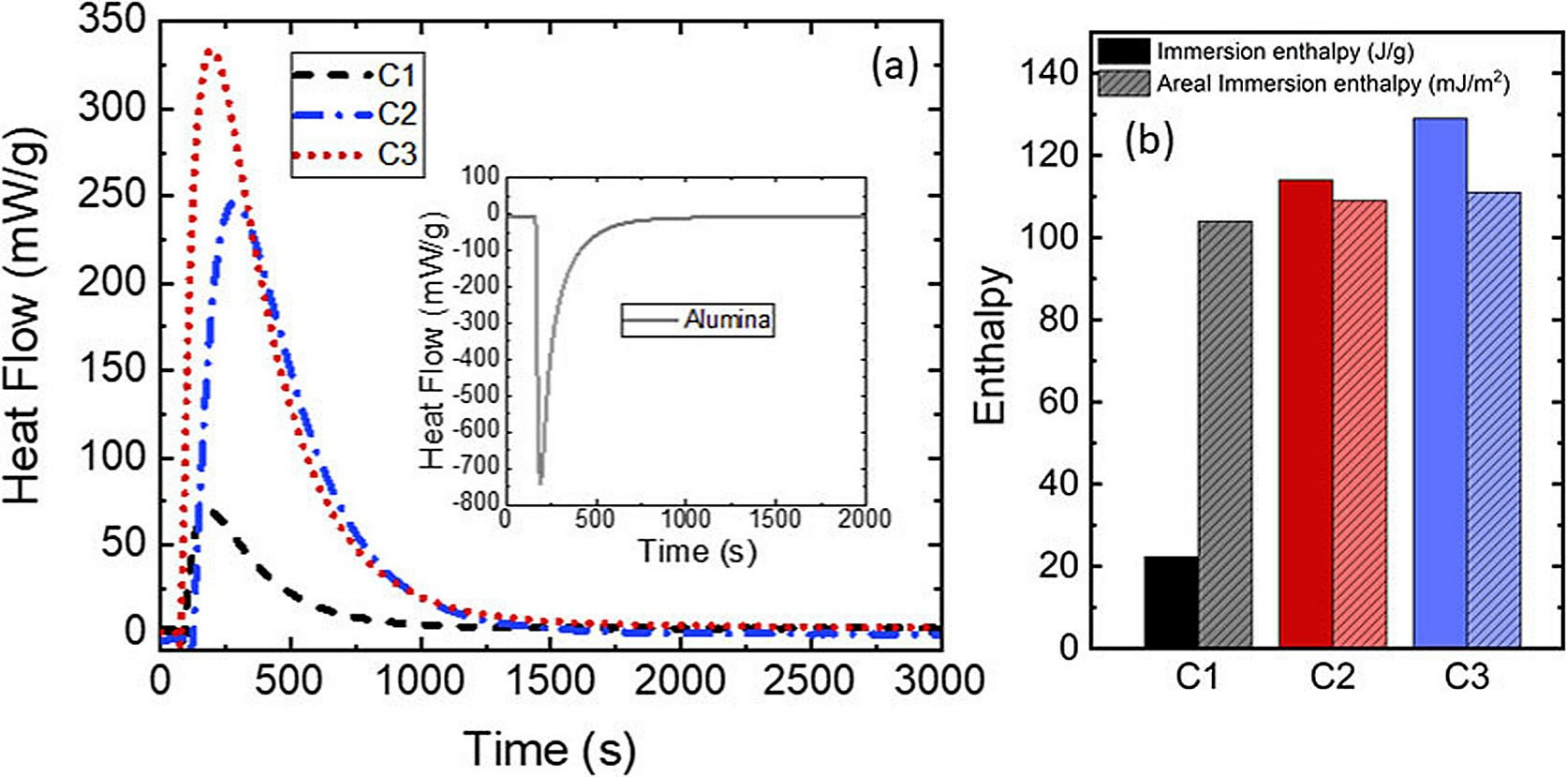
(a) Thermograms representing the heat flow as a function of time associated to the immersion phenomenon for the three porous carbons. For better legibility, the heat flow has been normalized to the mass of porous carbon. The immersion enthalpy is the integral of this curve. Inset: calibration by introducing a small piece of alumina at a different temperature from the cell. (b) Immersion enthalpy (J/g) and areal immersion enthalpy (mJ/m^2^) deduced using BET area.

Our measurements also demonstrate that the pore size range of the tested samples does not modify strongly the kinetics of wetting. This is an important result since all the S@C composites synthesized up to now by liquid impregnation are generally heated for 6 h up to 24 h. The immersion enthalpies in J per gram of sample are given in Figure 4b. The values increase with the specific area of the carbon. This is a first indication that liquid sulfur, that mainly consists of rings and short chains at 413 K, is able to enter in the microporous volume. This is consistent with the molecular sizes calculated by DFT.^[18]^ Once renormalized to the BET surface area, the areal immersion enthalpies (in J per m^2^) are very similar for the three carbons (Figure 4b, Table S4). This indicates that dispersive forces between sulfur and the surface are the main driving force for the filling of pores. Indeed, if only London additive dispersion forces are considered, the enthalpy of immersion of a porous solid into a liquid is at first order proportional to the wetted surface area.^[9]^ This is easy to understand if one considers large pores: the enthalpy of immersion is the product of the surface area by the immersion enthalpy per unit area. If the pores are narrow, the situation may appear at first sight different since a molecule is interacting with more than one wall (for instance two in a slit shaped pore geometry).However, due to the additivity of London dispersion forces, it has been shown that when a molecule has a size close to the pore width, its interaction energy is twice the one obtained on an open surface but the molecule also wets twice as much surface area.^[9]^ In the case of a cylinder, it was also shown that the ratio of energies is close to the ratio of wetted surfaces.^[9]^ This simplified analysis was confirmed by DFT calculations on a large range of pore sizes.^[19]^ Finally, since the three types of carbon lead to the same immersion enthalpy per unit area for liquid sulfur, they have probably similar surface‐liquid interactions and the liquid wets the whole surface accessible to nitrogen. This is confirmed by the estimation of the accessible surface area to liquid sulfur (SI Table S4) using immersion enthalpy of C1 as reference.

It has been recently reported that chemical interactions between sulfur and carbon could be present.^[20]^ They do not seem to be predominant here, otherwise the areal immersion enthalpy of microporous carbons would have been different because they have a higher density of graphene sheet defects (Figure 2). The value of immersion enthalpy per unit area is close to the work of adhesion – around 0.1 J/m^2^ at 393 K derived from contact angle measurements.^[17]^

This study opens a path to the rationale of the impregnation of porous carbon by sulfur. Depending on the temperature of impregnation, it is possible to fill preferentially microporous domains *via* vapor impregnation, or both micro‐meso‐macro domains *via* liquid impregnation. Immersion calorimetry allows the evaluation of the energy of interaction of sulfur with the porous material and also to follow the kinetic of wetting, which is quite fast for the samples studied here. At this temperature, where mainly S_8_ rings are present in the liquid, micropores can be filled by sulfur and the total accessible surface area to liquid sulfur is similar to the one determined by nitrogen adsorption at 77 K.

## Experimental Section

**Transmission Electron Microscopy** (TEM) coupled with electron energy loss spectroscopy (EELS) analyses were performed in order to monitor the atomic and electronic structures of the porous carbon matrices. The measurements were performed using a FEI TITAN 80–300 TEM equipped with an image corrector and a GATAN TRIDIEM imaging filter. The carbon K‐edge spectra were acquired with an energy resolution of 0.8 eV.

**Thermogravimetric Analyses** (TGA) were done using a TGA/DSC 1 apparatus from Mettler‐Toledo. All measurements were performed under Nitrogen flow (30 ml/min) using a heating rate of 5 K/min from room temperature up to 1173 K. Firstly, TGA is used to determine the temperature at which the porous carbon matrices must be treated to ensure complete removal of residual water and adsorbed organic species. Secondly, it was used to perform thermo‐desorption on the S@C composite and thus to quantify the amount of sulfur in the composite and to highlight the specific interaction of sulfur with carbon.

**Nitrogen Adsorption** at 77 K analyses were done in order to access the total porous volume of the carbon matrices before and after impregnation by sulfur in order to follow the accessibility of the pores. Porous carbon matrices were treated at 423 K under a residual pressure lower than 1 Pa before adsorption during 15 hours. After impregnation, the samples were kept under vacuum at room temperature before the adsorption measurement. Experiments were carried out with a BELMax from BEL Instrument for empty matrices and using a GEMINI apparatus from Micromeritics for the composite since the amount adsorbed is quite low.

**Immersion Microcalorimetry** measurements were done using a Tian‐Calvet type isothermal microcalorimeter. The calorimeter was set at a temperature equal to 413 K in order to ensure that the sulfur is in the liquid state but has not yet polymerized. The details of the experiment are given in SI (section 5).

## Conflict of interest

The authors declare no conflict of interest.

## Supporting information

As a service to our authors and readers, this journal provides supporting information supplied by the authors. Such materials are peer reviewed and may be re‐organized for online delivery, but are not copy‐edited or typeset. Technical support issues arising from supporting information (other than missing files) should be addressed to the authors.

Supporting Information

## Data Availability

The data that support the findings of this study are available from the corresponding author upon reasonable request.
