# Supplementary material for: Energetics of Sulfur‐Carbon Interaction
Source: Chemphyschem. 2022 Nov 14;23(24):e202200416. doi: 10.1002/cphc.202200416 (PMC10100209; doi:10.1002/cphc.202200416)
Supplement: Supplementary file 1 — Supporting Information [file CPHC-23-0-s001.pdf]

ChemPhysChem

Supporting Information

Energetics of Sulfur-Carbon Interaction

Marie-Vanessa Coulet,* Loïc Gourmellen, and Renaud Denoyel

1- Starting Materials

Sulfur in powder form (purity 99.9999%) was purchased to Sigma Aldrich. Prior to the impregnation into the carbon matrices, the sulfur was purified by performing at least 3 cycles of melting-freezing under dynamic vacuum. This procedure aims to remove the dissolved gases (H2S, air) and the adsorbed water molecules.

Three commercial porous carbons have been used. Carbopack X (C1) is a mesoporous graphitized carbon provided by Sigma-Aldrich. The average pore size given by the manufacturer is 10 nm. The second carbon, C2, is a commercial activated charcoal commercialized by Sigma Aldrich under the name Norit. Finally, the last sample, C3, has been provided by Takeda Chemical Ind. Co. Ltd.

BET surface areas are determined by applying the BET method with a particular attention to satisfy Rouquerol's criterium [1] in the case of microporous carbon. Pore size distributions were determined by the quenched solid DFT method developed by Neimark *et al.*[2] and integrated in the Quantachrome software. Despite the approximations used in this method, it has been proved to reproduce quantitatively the adsorption isotherms and pore sizes of model samples. It is also a direct way to compare samples. In the present case, a slit pore model was used as it is usually done for carbon samples.

Table S1: BET surface area and porous volume obtained by nitrogen adsorption at 77 K.

	**BET surface area (m^2/g)**	**Porous volume (cm^3/g)**
**C1**	215	0.49
**C2**	1048	0.42
**C3**	1221	0.49

2- Water adsorption isotherms

Water vapor adsorption at 298K was performed using a BEL Japan BELSORP Max. Helium was used to estimate the dead volume prior to measurements. Adsorption equilibrium was assumed when the variation of the cell pressure was below 0.5% for a minimum period of 300 seconds. Prior to the measurements, the samples were activated to 423 K under secondary vacuum (lower than 1Pa) for 16 hours.

[1] F. Rouquerol, J. Rouquerol, K. S. W. Sing, P. L. Llewellyn, G. Maurin, *Adsorption by Powders and Porous Solids: Principles, Methodology and Applications*, Elsevier/AP, Amsterdam, **2014**.

[2] M. Thommes, K. Kaneko, A. V. Neimark, J. P. Olivier, F. Rodriguez-Reinoso, J. Rouquerol, K. S. W. Sing, *Pure and Applied Chemistry* **2015**, *87*, 1051–1069.

The obtained isotherms are given below:

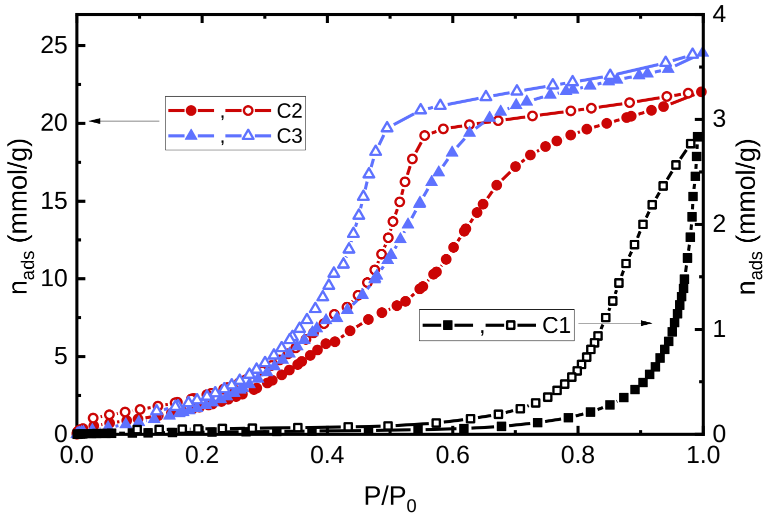

Figure S1: Water vapor sorption isotherms obtained for the three carbons. Full symbols are used for the adsorption branch and open symbols are used for the desorption branch

3- Mercury porosimetry

Mercury porosimetry experiments were carried out with the Poremaster apparatus from Quantachrome. Intrusion and extrusion were done after the sample was evacuated under vacuum. The cumulative intruded volume is measured as a function of intrusion pressure. This latter is transformed in pore size by applying the Laplace Washburn equation with a contact angle of 130°.

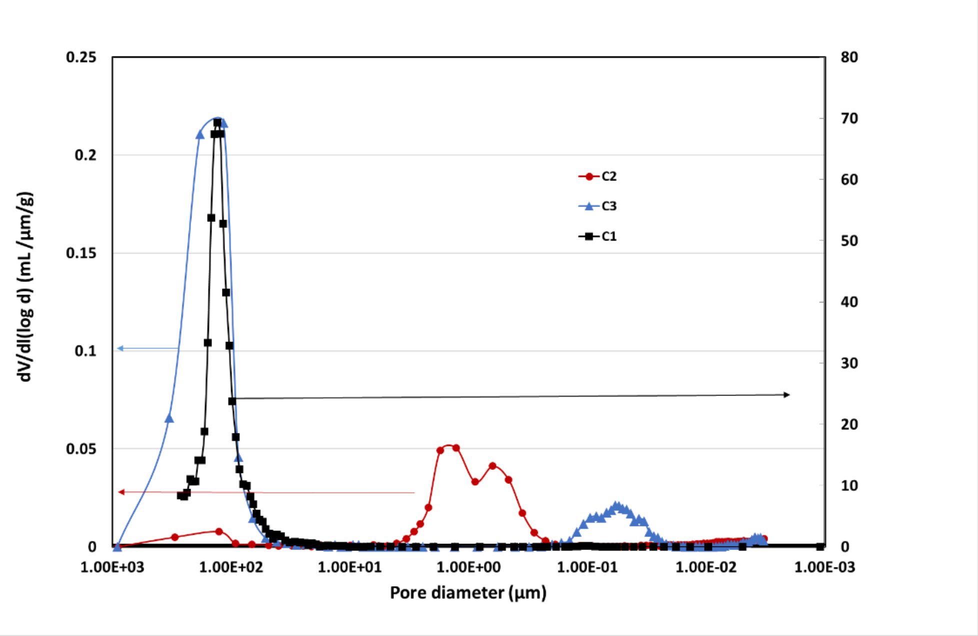

Figure S2: Pore size distribution obtained from mercury porosimetry.

4- Synthesis of sulfur@carbon composites

The carbon matrices were first heated under secondary vacuum up to 523 K for 5 hours to remove any adsorbed species. They were then transferred without breaking the vacuum into a glove box together with the purified sulfur. All the forthcoming handling of the samples was done inside the glove box. The samples were impregnated either from the liquid phase or from the vapor phase. For the liquid impregnation, pretreated carbon and purified sulfur were introduced in a glass tube (Fig. S3a). The tube was then sealed under vacuum and placed in an oven at 413 K. For vapor impregnation, an H-shape tubing system was used (Fig. S3b). Sulfur and carbon were put separately in the vertical tubes. Each side was sealed so that the whole H-cell is under vacuum. The H-cell was placed in a Nabertherm oven at 673 K for 12h. At this temperature, the vapor pressure of sulfur is reported to be around 50 kPa.

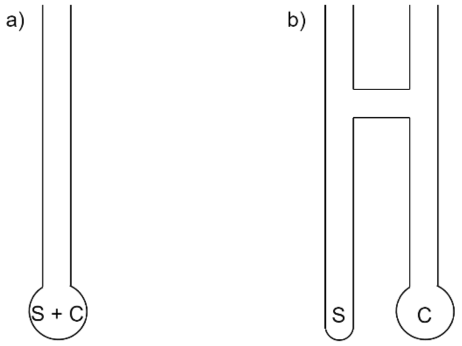

Figure S3: Sketch of the cells used for sulfur impregnation in the liquid phase (a) and in the vapor phase (b)

The initial masses introduced in the cells are given in Table S2. The as-prepared composites are denoted $S_L@C_x$ or $S_V@C_x$, where x is 1, 2 or 3 is for the porous carbon matrix (see above) and Land V stand for liquid or vapor impregnation.

Table S2: Initial masses of sulfur and carbon used to synthetize the composites by liquid impregnation (S_L@C) and by vapor infiltration S_V@C.

	S_L@C1	S_V@C1	S_L@C2	S_V@C2	S_L@C3	S_V@C3
**Initial mass of C (g)**	0.1505	0.203	0.1705	0.2074	0.1777	0.1751
**Initial mass of S (g)**	0.1483	0.2112	0.0935	0.1109	0.1583	0.1469
**Initial w%S**	50	51	35	35	47	46
**w%S from TGA**	47	40	32	32	43	38

Table S3: BET area and total porous volume of the carbon matrices before and after sulfur impregnation.

	**BET surface area (m^2/g)**	**Porous volume (cm^3/g)**
**C1**	215	0.49
**S_L@ C1**	2	0
**S_V@C1**	31	0.03
**C2**	1048	0.42
**S_L@C2**	2	0
**S_V@C2**	24	0.003
**C3**	1221	0.49
**S_L@C3**	27	0.003
**S_V@C3**	45	0.008

5- Immersion microcalorimetry

The interaction of sulfur with the carbon samples was studied by immersion microcalorimetry using a Tian-Calvet type isothermal microcalorimeter. The calorimeter was set at a temperature equal to 413 K in order to ensure that the sulfur is in the liquid state but has not yet polymerized. The sulfur is introduced into a cylindrical cell. The carbon sample, that has been treated at 423 K under secondary vacuum, is placed in a sealed bulb in glass with a brittle end. The bulb is suspended to a glass rod and introduced in the cylindrical cell containing the purified sulfur. The calorimetric cell is introduced in the calorimeter and let for stabilization overnight. The glass rod can be pushed down in order to break the brittle end at the bottom of cell. Once broken, the glass bulb is filled by liquid sulfur and this leads to the wetting of the sample. The heat obtained is then corrected for the heat produced by breaking an empty bulb and by the vaporization of sulfur obtained from an experiment without carbon inside the bulb. The resulting heat gives access to the enthalpy of immersion. The calibration constant of the calorimeter at 413 K is evaluated by measuring the heat produced when an α-alumina ingot is introduced in the calorimeter. The alumina is dropped from outside the calorimeter with an initial temperature of 313 K. The tabulated specific heat values between 313 K and 413 K were used to obtain the calibration constant.

Table S4: Enthalpies derived from immersion calorimetry. In the second column the energy is reported to BET surface area. The accessible surface area is determined using C1 as a reference. It is obtained by multiplying the reference BET surface area by the ratio of immersion enthalpies, C2/C1 or C3/C1.

	**Immersion enthalpy $\Delta H_{immersion}$ (J/g)**	**Areal Immersion enthalpy $\Delta H_{immersion}$ (mJ/m^2)**	**Accessible surface area to sulfur (m^2/g)**
**C1**	22.3 ± 0.7	104 ±5	--
**C2**	114 ± 1	109 ± 1	1099 ± 30
**C3**	129 ± 3	111 ± 3	1244 ± 40
